# Hyperbilirubinemia as a Possible Predictor of Appendiceal Perforation in Acute Appendicitis: A Prospective Study

**DOI:** 10.7759/cureus.21851

**Published:** 2022-02-02

**Authors:** Sibabrata Kar, Tapan K Behera, Kumaramani Jena, Ashok Kumar Sahoo

**Affiliations:** 1 Surgery, SCB (Srirama Chandra Bhanj) Medical College and Hospital, Cuttack, IND; 2 Surgery, Pandit Raghunath Murmu Medical College and Hospital, Baripada, IND; 3 Neurosurgery, SCB (Srirama Chandra Bhanj) Medical College and Hospital, Cuttack, IND; 4 Surgery, Jawaharlal Institute of Postgraduate Medical Education and Research (JIPMER), Puducherry, IND

**Keywords:** histopathology, hyperbilirubinaemia, total leukocyte count, total serum bilirubin, acute appendicitis

## Abstract

Introduction: Acute appendicitis, a common abdominal surgical emergency, can mostly be diagnosed clinically by assessing the symptoms and physical findings but confirmation of the diagnosis solely depends upon the histopathological study of the resected appendix specimen, being supplemented by a few laboratory tests and ultrasonography (USG). In spite of all these available investigations, the diagnosis of acute appendicitis, because of its nebulous presentation and the variability of signs, remains a Herculean task for the surgeon.

Methods: This is a prospective study conducted on 125 patients diagnosed with acute appendicitis and posted for appendicectomy. Total serum bilirubin (TSB), and total leucocyte count (TLC) were done in all cases. USG of the abdomen was done in all the cases to confirm the diagnosis and to rule out other causes of acute abdomen. TLC more than 11 x 10^3^ cells/µL and TSB more than 1.1 mg/dL were considered positive. They were operated on and their diagnoses were confirmed post-operatively by histopathological examination. Patients were subdivided according to histopathological findings into: normal appendix (n = 11), uncomplicated acute appendicitis (n = 86), gangrenous appendicitis (n = 10) and perforated appendix (n = 18). Laboratory results, operative findings, and histopathological findings were compiled, analyzed, and compared with reference values.

Results: Out of 125 patients, 114 (91.2%) were histologically positive for acute appendicitis, while 11 (8.8%) had normal histology. TLC was elevated in 95 (76 %) patients and it was normal in 30 (24%) cases. Among the patients with leucocytosis, only 90 (94.74%) had positive histology for acute appendicitis, while the remaining five (5.26%) had normal histology. Among the 30 patients who had normal TLC, 24 had positive histology for acute appendicitis, while the remaining six had normal histology. The specificity, sensitivity, positive predictive value (PPV), and negative predictive value (NPV) were 78.95%, 54.55%, 94.74%, and 20%respectively. Similarly, 67 patients (53.6%) had elevated TSB, while it was within normal limits in 58 (46.4%) patients. From the patients with hyperbilirubinemia, 65 (97.01%) had positive histology for acute appendicitis, while the remaining two (2.99%) had normal histology. Among 58 patients who had normal TSB, 49 had positive histology for acute appendicitis, while the rest nine had normal histology. The specificity, sensitivity, PPV, and NPV are 57.02%, 81.82%, 97.01%, and 15.52% respectively. However, when both TLC and TSB were compared as markers of appendicular perforation, the sensitivity, specificity, PPV, and NPV of total serum bilirubin were found to be 89.29% against 21.43%; 53.49% vs. 2.33%; 38.46% vs. 6.67% and 93.88% vs. 8.33% of total leukocyte counts respectively.

Conclusion: Elevated total serum bilirubin could be used as a better predictor of appendiceal perforation in acute appendicitis.

## Introduction

One of the most common surgical problems encountered in the emergency department by a surgeon is appendicitis [1,2]. In most cases, the diagnosis is made by the combinations of signs and symptoms of the patients. However, atypical presentations are not rare as around half of the patients diagnosed with acute appendicitis may not actually have the disorder thus resulting in 22%-30% negative appendectomies [3-6]. Conversely, a diagnostic delay can land a patient onto complicated appendicitis with higher morbidity and mortality.

To minimize negative appendectomies, various blood investigations (white blood cell counts, C-reactive protein) and radiological investigations (ultrasonography, computed tomography scan) have been analyzed [7-13]. Various scoring systems like the Alvarado score and the Modified Alvarado score, taking clinical features and laboratory investigations into account, have also been designed to diagnose this malady [14-16]. Recently, elevation in total serum bilirubin has been investigated as a marker for the diagnosis of acute appendicitis and a predictor of appendiceal perforation in patients of acute appendicitis [17].

It is a well-known fact that leukocytes are the main line of defense for any invasion by microbes. Therefore, any microbial infection will lead to an increase in total leucocyte count. Transmigration of bacteria and subsequent release of TNF-alpha, interleukin(IL)-6, and other cytokines occurs when bacteria invade the appendix. Hepatic dysfunction can occur directly through the superior mesenteric vein causing inflammation or abscess formation. Indirectly reshuffling the blood flow to the liver can cause hepatic dysfunction [18-24]. So, it has been established that hyperbilirubinemia has a definitive role in appendicular pathology. Hence, the study was conducted to evaluate the role of hyperbilirubinemia as a predictive factor for appendiceal perforation in acute appendicitis.

## Materials and methods

The study was conducted in one of the tertiary care hospitals in western Odisha from September 2010 to August 2012. The study was approved by the Institutional Ethics Committee of Veer Surendra Sai Institute of Medical Sciences and Research, Sambalpur, Orissa with an approval number IEC/IRB No: 188/2010 and has been performed following the ethical standards laid down in an appropriate version of the Declaration of Helsinki (as revised in Brazil 2013). It was a prospective study comprising 125 patients with the diagnosis of acute appendicitis and planned for an emergency appendectomy. All the consecutive patients with acute appendicitis were included in the study. The diagnosis of acute appendicitis was made by senior experienced consultants clinically based on the signs and symptoms (symptoms - migratory right iliac fossa pain, nausea and vomiting, anorexia, fever, burning micturition, loose stool; and signs - peritoneal inflammation like right iliac fossa tenderness, rebound tenderness and localized guarding, tachycardia). The patients who had comorbid conditions, patients who were managed conservatively, patients admitted for interval appendicectomy following recurrent appendicitis or appendicular mass treated conservatively previously, patients with liver diseases, hemolytic diseases, or other congenital or acquired biliary diseases, and patients with alcoholism history were excluded from the study.

Patients with clinical suspicion of acute appendicitis were subjected to further investigations as per the institutional protocol. Urinary microscopic examination was performed in all cases. Chest X-ray, echocardiography, and ECG were done according to the necessity of the patient. The blood investigations which were done in all the patients were total serum bilirubin (TSB), total leucocyte count (TLC), and differential count. TSB of more than 1.1 mg/dL was considered to be positive. A TLC of more than 11 x 10^3^ cells/µL was leveled as positive. The sample was collected by standard approved technique without any special preparation for the patients. In case of delay, the sample was stored at 2-8 °C up to a maximum of 72 hours. To rule out other causes of acute abdomen and to substantiate the clinical diagnosis of acute appendicitis, ultrasonography (USG) of the abdomen was done in all the cases.

After all the investigations, if the patient was found to have acute appendicitis, then an emergency appendectomy was done after getting proper consent. The appendectomy specimen was subsequently sent for postoperative histopathological examination. The final diagnosis was established by the histopathology report. According to the histopathological report, the patients were divided into four groups. Such as patients having normal appendix (n = 11), uncomplicated acute appendicitis (n = 86), gangrenous appendicitis (n = 10) and perforated appendix (n = 18).

The patients were considered as true positive if they had hyperbilirubinemia. False-positive were those cases who were negative in histopathology but had hyperbilirubinemia. Similarly, the histopathologically positive patients among the hyperbilirubinemia negative group were considered false negatives. Accordingly, true negatives were having neither hyperbilirubinemia nor having any features of appendicitis histopathologically. In the same manner, TLC and USG abdomen were also classified as true and false positives and true and false negatives after correlating it with histopathology reports.

All the blood investigation reports, intraoperative findings, and the final histopathological report were put together, interpreted, and equated with the reference values. The assessment of TSB and TLC in diagnosing acute appendicitis and appendicular perforation was done using the 2x2 contingency table (Table 1). By using the table, specificity, sensitivity, PPV, and NPV were calculated.

**Table 1:** 2x2 contingency table used to calculate sensitivity, specificity, PPV and NPV HPE = Histopathological examination; PPV = Positive predictive value; NPV = Negative predictive value; TSB =Total Serum Bilirubin; TLC =Total Leucocyte Count a: True Positive; b: False Negative; c: False Positive; d: True Negative; n: total number of samples

Test (TSB/TLC)	HPE	
	Positive	Negative
Positive	a	b
Negative	c	d

In Table 1 'a' denotes true positive. That means if the table has been used for TLC, then 'a' will represent patients who have both high TLC and appendicular perforation on HPE. Similarly, 'd' denotes true negative. That means if the table has been used for TLC, then 'd' will represent patients who have neither high TLC nor appendicular perforation on HPE. Symbols 'b' and 'c' denote false positive and false negative respectively. False-positive signifies that the patient has high TLC but not appendicular perforation on HPE. Similarly, false negative symbolizes that the patient has an appendicular perforation on HPE but the TLC is not high. Similar calculations had been done using TSB and HPE. All the above explanations have been summarized through the formulae given below.

Sensitivity = (a/a+c) x 100

Specificity = (d/b+d) x 100

Positive Predictive Value (PPV) = (a/a+b) x 100

Negative Predictive Value (NPV) = (d/c+d) x 100

All the patients were followed up in the outpatient department every two weeks for a period of eight weeks. During follow-up, they were enquired about the quality of life through a standard set of questionnaires and examined to detect the occurrence of late complications.

## Results

According to the inclusion criteria, a total of 125 patients were eligible to participate in the present study. The participants belonged to varying age groups from four to 70 years with a mean age of 30.76 ± 14.09 years. In the present study, the incidence of acute appendicitis was marginally higher in females (M:F = 1:1.15) while it was males who were affected more with appendicular perforation (M:F = 3:1). The age groups which were affected more in acute appendicitis and appendicular perforations were 21-40 years (40%) and 21-30 years (7.2%) respectively (Table 2).

**Table 2:** Distribution of age and gender of acute appendicitis and appendicular perforation (n =125)

Age group (years)	Acute appendicitis			Appendicular Perforation		
	M	F	Total	M	F	Total
<10	0	1	1	0	1	1
11-20	6	10	16	3	1	4
21-30	15	12	27	7	2	9
31-40	10	13	23	1	1	2
41-50	6	7	13	7	1	8
51-60	3	3	6	2	1	3
>60	0	0	0	1	0	1
Total	40	46	86	21	7	28

**Figure 1:**
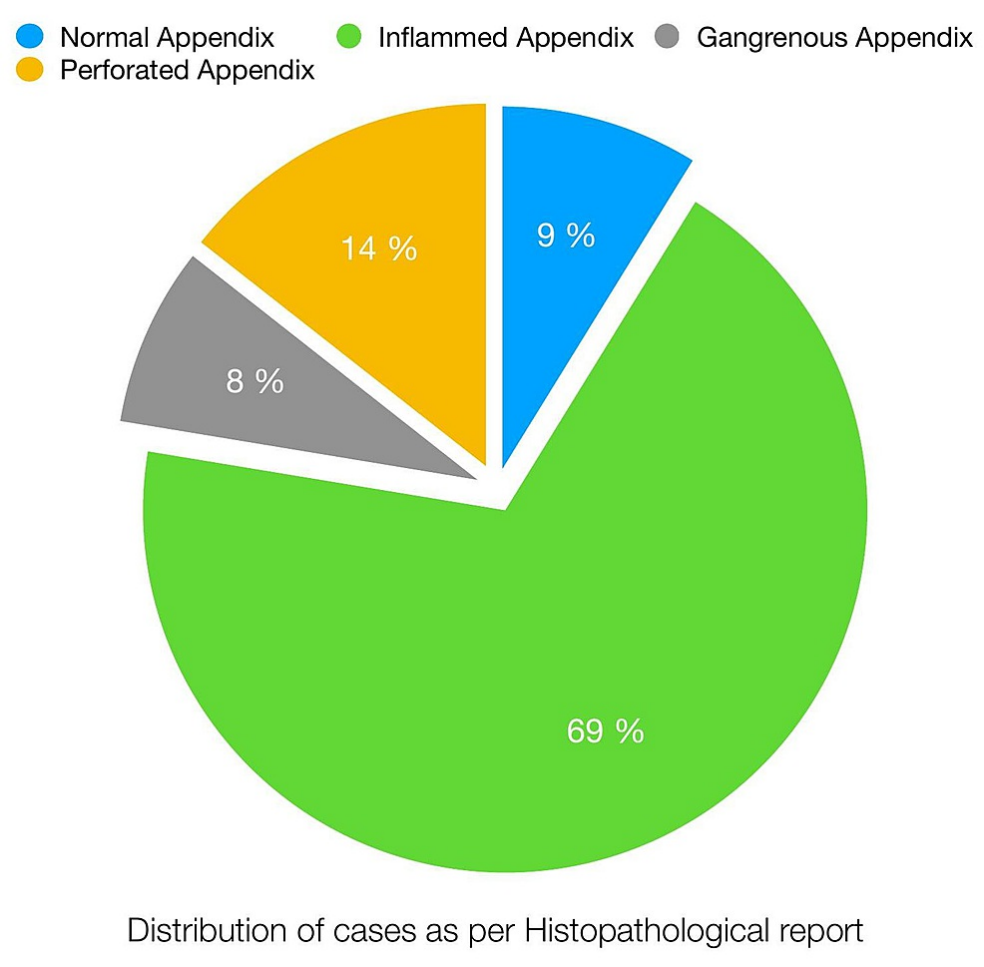
Distribution of patients as per Histopathological report

Among the 125 patients, 114 patients had a histopathological diagnosis of acute appendicitis, whereas the rest of the patients (11) had normal histology (Table 3, Figure 1).

**Table 3:** Classification of patients according to the level of TSB and TLC (n = 125). TSB =Total Serum Bilirubin; TLC = Total Leukocyte Count

Type of Appendix on histo-pathology	Total Serum Bilirubin		Total Leukocyte Count	
	< 1.1 mg/dL	> 1.1 mg/dL	< 11 x 10^3^cells/µL	> 11 x 10^3^cells/µL
	No (%)	No (%)	No (%)	No (%)
Acute Appendicitis	46 (36.8%)	40 (32%)	2 (1.6%)	84 (67.2%)
Gangrenous Appendix	2 (1.6%)	8 (6.4%)	8 (6.4%)	2 (1.6%)
Perforated Appendix	1 (0.8%)	17 (3.6%)	14 (11.2%)	4 (3.2%)
Normal Appendix	9 (7.2%)	2 (1.6%)	6 (4.8%)	5 (4%)
Total	58	67	30	95

Ninety-five (76%) patients had an elevated TLC whereas it was normal in the rest (30) of the patients. Ninety (94.74%) patients were with elevated TLC had histologically acute appendicitis, while the remaining five (5.26%) had normal histology. Thirty (24%) patients had normal TLC, 24 of them had positive histology for acute appendicitis, while the remaining six had normal histology. The specificity, sensitivity, positive predictive value (PPV), and negative predictive value (NPV) were 78.95%, 54.55%, 94.74%, and 20% respectively. Similarly, total serum bilirubin was elevated in 67 patients (53.6%), while it was within normal limits in 58 (46.4%) patients. Both elevated total serum bilirubin and positive histology were found in 65 (97.01%) patients while two patients had normal histology with elevated total serum bilirubin. Among 58 patients who had normal total serum bilirubin, 49 of them had positive histology for acute appendicitis, while the rest nine had normal histology. The specificity, sensitivity, PPV, and NPV are 57.02%, 81.82%, 97.01%, and 15.52% respectively as shown in Table 3.

However, when both TLC and TSB were analyzed as predictors of appendicular perforation, the sensitivity, specificity, PPV, and NPV of total serum bilirubin were found to be 89.29% against 21.43%; 53.49% vs. 2.33%; 38.46% vs. 6.67%, and 93.88% vs. 8.33% of total leukocyte count respectively (Table 4).

**Table 4:** Comparison between Total Serum Bilirubin and Total Leukocyte Count as markers of Appendicular Perforation PPV = Positive Predictive Value; NPV = Negative Predictive Value

Laboratory value	Sensitivity (%)	Specificity (%)	PPV (%)	NPV (%)
TLC > 11 x 10^3^ cells/µL	21.43	2.33	6.67	8.33
Total Bilirubin > 1.1 mg/dL	89.29	53.49	38.46	93.88

## Discussion

Uncomplicated acute appendicitis can be managed either by conservative management or by appendectomy without taking a long time for recovery. But in the case of complicated appendicitis such as appendicular perforation, the management is not so straightforward. Similarly, the recovery also takes a longer time than usual after surgery. Some of the patients may develop various kinds of morbidity; in rare cases, mortality also. Various modalities have been researched to anticipate at the earliest to diagnose or suspect a case of appendicular perforation. So that the management and recovery will become uncomplicated. Among those various study topics, TSB is one of them which had been explored in the present study. In the present study, the postoperative histopathology found 91.2% of patients had acute appendicitis, in whom the TLC and TSB were raised only in 76% and 53.6% of cases respectively. From the above result, it's prudent that there is no linear relationship between the elevation of both TLC, TSB, and the acute inflammation of the appendix. A similar result had been produced by a study done in Nepal by Khan S [25].

In the present study, the sensitivity, specificity, PPV, and NPV of WBC in uncomplicated acute appendicitis was 78.75%, 80%, 94%, and 48.48% respectively. Similarly, the sensitivity, specificity, PPV, and NPV of TSB were 57.02%, 81.82%, 97.01%, and 15.52% respectively. Also, this study revealed that the sensitivity, specificity, PPV, and NPV of TSB in differentiating perforated or any complicated appendicitis from uncomplicated appendicitis was 89.29%, 53.49%, 38.46%, and 93.88% respectively, whereas it was 21.43%, 2.33%, 6.67%, and 8.33% respectively for TLC. According to the above results, it can be ascertained that TSB can be a better predictor of perforation in acute appendicitis than TLC. As a consequence, TSB should be included in the evaluation of patients with suspected appendicitis. A similar result was obtained by another study conducted by Chaudhary et al. [26].

The role of raised bilirubin has not been analyzed properly in the preoperative workup for appendicular perforation. The possible explanation for hyperbilirubinemia in the case of a perforated appendix might be due to excessive production of bilirubin and derangement in its hepatic clearance [27].

The causative factor for the decayed hepatocellular function in septic patients might be the micro-organism, its toxin, or the cytokines they release. The pathway of spread from the inflamed gastrointestinal tract is through either the portal vein or the lymphatics. Various studies demonstrated that bacterial infection can lead to derangement in the production of bile acid and its circulation. The end result is hyperbilirubinemia in septic patients. Consequently, these patients along with the patients with an extrahepatic bacterial infection manifested as cholestasis which usually is induced by nitric oxide and proinflammatory cytokines produced by hepatic and ductal bile formation [27].

Most of the patients with acute appendicitis harbor *E. coli* and *Bacteroides fragilis* in their walls [28,29]. These insulting organisms cause the inhibition of hepatic microcirculation and consequently damage the sinusoids. The lipopolysaccharides associated with *E. coli *are the main culprit that deranges the hepatic uptake and bile acid secretion. *E. coli* infection also causes continuous hemolysis of RBCs leading to raised bilirubin levels. These all may cumulatively lead to hyperbilirubinemia [27].

In the initial stage of sepsis, though the cardiac output and hepatic blood flow increased along with decreased peripheral resistance, it cannot prevent hepatocellular dysfunction [30]. The resultant hepatocellular dysfunction is the consequence of elevated levels of circulating pro-inflammatory cytokines such as TNF and IL-6 [24]. It concludes that the accentuated levels of TNF-alpha and IL-6 may be the inciting agent for the hepatocellular dysregulation in the early stage of sepsis.

From the above discussion, it can be ascertained that in the day-to-day practice of a surgeon, a male patient with suspected acute appendicitis having leucocytosis and hyperbilirubinemia should be managed in the line of management of perforated appendicitis.

The limitations of the present study are that it is a single-center study and the sample size is small.

## Conclusions

In the present study, it was found that hyperbilirubinemia can be used as a strong adjunctive investigation in the early diagnosis of appendicular perforation and its management which can prevent the morbidity and mortality arising from the same. The study demonstrated that triple assessment in the form of clinical evaluation (signs and symptoms), laboratory investigations (total serum bilirubin and total leucocyte count), and the imaging modalities (ultrasonography, computed tomography) would definitely diagnose a complicated and uncomplicated appendicitis.

This small study can be used in the management of suspected perforated appendicitis by adding a single, simple blood investigation i.e. total serum bilirubin. Though it cant be generalised due to the small sample size, further studies are needed to establish the conclusion of the study.
